# Characterization of exoplanets in the James Webb Space Telescope era

**DOI:** 10.1073/pnas.2507109122

**Published:** 2025-09-22

**Authors:** Jonathan I. Lunine, Neta Bahcall

**Affiliations:** ^a^Jet Propulsion Laboratory, California Institute of Technology, Pasadena, CA 91109; ^b^Division of Geological and Planetary Sciences, California Institute of Technology, Pasadena, CA 91125; ^c^Department of Astrophysical Sciences, Princeton University, Princeton, NJ 08544

“There are therefore innumerable suns, there are an infinite number of earths that similarly go around those suns, just as the seven we see go around this sun near to us… we see the suns, which are the largest, indeed immense, bodies, but we do not see the earths which, being much smaller bodies, are invisible.”^*^
Giordano Bruno, *On the Infinity of the Universe and the Worlds,* 1584

Over four centuries ago, the Italian philosopher Giordano Bruno reasoned qualitatively but correctly why detecting and characterizing planets around other stars would be so difficult (1). It would not be until 1995—30 years ago—that the first planet around another main sequence star was discovered, albeit indirectly (2). Direct sensing of an Earth-sized planet at 1 astronomical unit (AU) around a Sun-like star is still out of reach of even the most advanced coronagraphic systems for suppressing the light of the planet’s parent star (3). Nonetheless, in the time between 1995 and today, a revolution has occurred in planetary science thanks to the ability to determine the existence, size, mass, and orbital parameters of thousands of planets around other stars, and the composition of major species in the atmospheres of roughly 100 such objects (4). The latest step in that revolution was the launch on Christmas Day 2021 of the James Webb Space Telescope (JWST), a space-based infrared observatory with a 6.5-meter primary mirror and state-of-the-art instrumentation cooled to temperatures as low as 7 degrees above absolute zero. JWST has obtained spectra of exoplanets with radii nearly as small as that of Earth, around M-dwarf stars (5), and found signatures consistent with atmospheres around planets 2.5 times the size of Earth (6). (From here on, we use the term “planet” interchangeably with exoplanet unless there is ambiguity with respect to whether we are referring to our solar system).

In this special feature, five articles document some of the exciting results obtained by JWST about exoplanets and describe the theoretical underpinnings of their analysis and interpretation. Two of these papers (5, 7) focus on observations of planets that are, respectively, Earth- and Jovian-sized, while a third focuses on the enigmatic “sub-Neptune” class of planets for which no known equivalent exists in our solar system (8). A fourth paper (9) considers JWST as a tool for diagnosing other aspects of planets around other stars: possible rings, moons, and the dynamical states of their orbits, while the fifth (10) details the promise and pitfalls in using JWST data in the search for life-bearing planets and life itself.

Although JWST can detect bright and therefore large planets in distant orbits, by blocking the light of the parent star using an optical device called a coronagraph, most planets—and all those detailed in this special feature—are detected via the transit technique. When the planet passes in front of the star, blocking part of the stellar disk, the transit is “primary”; conversely when the planet is observed as it passes behind the star it is called a “secondary” transit. Spectra collected during primary transit are synthesized from the variation in the apparent size of the planet as a function of wavelength. These are transmission spectra caused by the variation in opacity of the planet’s atmosphere by gas or grains, as a function of wavelength. During the secondary transit, a spectrum is obtained of the star plus the planet at nearly full phase; the stellar spectrum alone is obtained when the planet passes behind the star. Subtracting one from the other yields the planetary spectrum. This thermal emission spectrum is available whether the planet has an atmosphere or not. It is the revolutionary sensitivity of JWST, combined with its broad wavelength coverage and stability of its detectors, that makes possible the observation of spectra for planets down to the size of Earths around the smallest and coolest stars (5). Because transits happen only for very restrictive alignments of planetary orbits with the line of sight to the Earth, and the alignment is more restrictive for increasing size of the planetary orbit, almost all transiting planets are in very close orbits (less than a 10th of the Earth–Sun distance, or 0.1 AU) around their parent stars.

## Jovian-Sized Exoplanets

Probing of the atmospheres of giant planets around other stars began in 2001 with HD209458b, the first detected transiting exoplanet (11). In the case of stars similar in mass and hence luminosity to the Sun, which harbor most of the giant planets known to exist, the proximity to the star creates temperatures at the surface of the planet of thousands of degrees Kelvin. Hubble Space Telescope, Spitzer Space Telescope, and large ground-based facilities like Keck have provided spectra of these transiting planets that have revealed water and a few other molecules (12), but with signal-to-noise ratio (S/N) that has made quantitative abundance determinations difficult. The era of JWST is changing that. Wiser et al. (7) describe JWST observations of a giant planet, Wasp-80b, about half the mass of Jupiter and orbiting a cool K-dwarf about 9% the luminosity of the Sun. It is a relatively rare target because there are few such giant planets in orbit around low-mass and hence low-luminosity stars. It therefore has only a modest dayside temperature of about 820 K in contrast to most observed close-in giant planets which orbit intrinsically brighter stars. Consistent with a modest overall temperature of a few hundred Kelvin, the planet’s spectrum exhibits with very high confidence all the major light carbon species: methane, carbon monoxide, carbon dioxide, as well as water, and tentatively ammonia. In turn, this makes possible an accurate determination of the abundance of the major elements heavier than hydrogen and helium (its so-called metallicity).

While the star’s metallicity is like that of the Sun or slightly below, the planet has a supersolar metallicity, consistent with addition of solids into the planet during and possibly after formation. But the carbon-to-oxygen ratio, C/O, is below solar, suggesting that whatever solids were added were relatively poor in carbon-bearing compounds. While the details of what this means in terms of formation location (relative to condensation lines, or “ice” lines, in the protoplanetary disk, pebble vs. planetesimal accretion, etc.) are best left to a separate and more extended discussion, the article illustrates how JWST is making possible the extraction of scientifically interesting compositional data on gas giant planets with unparalleled S/N.

## Sub-Neptunes

As one proceeds downward in mass and therefore size from Jupiter, planets become more difficult to observe. But they also may become more informative of formation processes; Uranus and Neptune are far more enriched in heavy elements than Jupiter or Saturn, providing a better peek at the original solids in the protoplanetary disk. These planets are also complicated in having multiple phases and layers of water and rock in their interiors. Planets between the size of Neptune and Earth, called sub-Neptunes, are both the most abundant exoplanets in the known population and the most enigmatic. Population studies of sub-Neptunes yield a bimodal distribution of radii, perhaps indicating two distinct classes of planets with different formation mechanisms. As Madhusudhan and colleagues point out in their paper (8), there might be in fact three distinct kinds of planets represented in this radius range: smaller versions of Uranus and Neptune with extensive interior oceans, or mostly rocky planets with extended hydrogen-helium envelopes, or water worlds with a small rock core, extensive ocean, and a thin hydrogen rich atmosphere. This last possibility represents a class of planet, which they refer to as a Hycean world, with no counterpart in our solar system. And any of these are consistent with a given mass and radius. Therefore, the only way to distinguish among them is by determining their atmospheric compositions and then comparing these to predictive models. Because of their small size (1.5 to 4 times the radius of the Earth), obtaining the necessary spectra at sufficient S/N was not practical prior to the era of JWST.

Madhusudhan et al. (8) survey JWST spectra of sub-Neptunes to date and explain how comparison to models is yielding constraints on the nature of these planets. JWST’s capabilities enable high S/N detection of major species in sub-Neptune atmospheres. Clouds remain a problem, as they can diminish the strength of molecular features and contribute to ambiguity in comparing with models, but some observations are yielding impressive results. An outstanding example is K2-18b, for which JWST transmission spectra reveal methane and carbon dioxide with high confidence. Together with the nondetection of ammonia and carbon monoxide, the abundances of these species when combined with chemical models rule out mini-Neptunes or rocky worlds with hydrogen-rich atmospheres. This makes K2-18b perhaps the best candidate for a Hycean world and an intriguing one given that the planet’s distance from its cool M-dwarf star implies an equilibrium temperature (that in direct energy balance with the stellar insolation) similar to or modestly lower than that of Earth (8).

## Small Rocky Worlds

Exoplanets with a mass-radius combination indicating that they are made mostly of rock and metal, compositionally like and roughly the size of Earth, present the greatest challenge to JWST’s capabilities given their small size. Atmospheric composition of rocky planets around sun-like stars is beyond the reach of JWST, but not so for such planets around the smallest stars. There, an outstanding question that can be addressed with JWST, as Kreidberg and Stevenson point out in their paper (5), is whether such planets possess atmospheres at all. Airless planets need not have been formed that way; they might have lost their atmospheres through escape energized by absorption of stellar extreme ultraviolet radiation (EUV) early in a given system’s history, or by a high rate of impacts by large bodies. In the case of the former, as Kreidberg and Stevenson discuss, there may be combinations of EUV fluence and planetary escape velocity for which atmospheres of a given molecular weight would be unstable. Therefore, determining whether the planets in a given system have atmospheres or not is a clue to the early history of the star itself.

There are multiple interfering effects, including the presence of clouds, and detection of water vapor resident in cool starspots in the host star itself. To date, JWST has made no definitive detection of an atmosphere around any of the 10 rocky planets for which transmission spectra have been published (7). Thermal emission spectra are also consistent with no thick, CO_2_ -rich atmospheres for the planets observed in this way, although model atmospheres more like Earth’s cannot be ruled out. These are difficult observations, at the limit of JWST’s capabilities, but are of keen interest: a special Director’s Discretionary Time program will expand the envelope of observations of such planets, including objects with lower surface temperatures than observed to date (7).

## Dynamics

Two other papers discuss the application of JWST data to problems of keen interest in extrasolar planetary science. Millholland and Winn (9) describe the various dynamical phenomena inherent in exoplanet systems that can be diagnosed with JWST observations. The decay of the orbits of close-in Jupiters, subject to various dissipative interactions with their host stars, can be detected through the subtle and progressive decrease in period of the orbit and hence change in the timing and duration of transits over time. Tidal distortion of the shape of an exoplanet might be detectable in very favorable cases: Planets on very close orbits thermally inflated by the host star. Detection of oblateness caused by rotation is possible in a larger number of cases. The distribution of molecular abundances in a planet’s atmosphere, seen in JWST spectra, might also reveal additional heating through tidal interactions with the host star, although this is very much model-dependent.

Besides tidal phenomena, the presence of rings or moons around planets is of interest because such features give clues to the dynamical evolution of planets and, in the case of moons, might point to where life could exist in such distant systems. Distinguishing rings around a normal-sized planet versus the large radius of a thermally puffed-up planet is a challenge, and ring detectability in general depends on several factors. Detection of moons the size of Ganymede in our own solar system is also challenging with JWST, being possible only for giant planets around stars much smaller than the Sun. Whether substantially larger moons (e.g., the size of Earth) might be possible around giant planets is speculative, given that Ganymede (the size of Mercury) is the largest moon in our solar system.

## Search for Life

Seager et al. (10) provide a critical, observationally grounded view of the ability of JWST to detect the signatures of a planet capable of supporting life (“habitable”) and of potential life signs themselves (“biosignatures”). Assuming an achievable but still ambitious noise floor, expressed in terms of the precision of JWST’s photometry, of 30 ppm, they assess what combination of planetary and stellar sizes can produce detectable molecular signatures. Earths around stars like the Sun present too small a signal by far for JWST, and even Earths around the smallest, M dwarf, stars are a challenge. Sub-Neptunes—planets somewhat smaller than Uranus and Neptune—might be both habitable and large enough to allow detection of key atmospheric gases. Detection of a given gas is not, however, sufficient: One must find enough molecules to avoid ambiguities in the characterization of the planet from spectral data. Seager et al. give a case in point: Detecting water and methane in an exoplanet atmosphere can lead to multiple interpretations. Detection of biosignatures requires even more care, as the implications of the claim of life would be momentous. Seager et al. suggest that three criteria be applied to the claimed detection of a biosignature. First, the signal must be strong, that is, have a high S/N. Second, the identification of a given spectral feature with a particular gas must be unequivocal. Faint spectral features, or only a single feature where several should appear, may mislead and hence impede correct identification. Finally, the spectral identifications in terms of specific molecules must also be consistent with what is known about the planet; a planet whose atmosphere is too hot for stable methane ought not to have detectable amounts of methane.

Seager et al. close their paper with an analysis of the current controversy surrounding the reported detection in the atmosphere of K2-18b of a potential biosignature gas, dimethyl sulfide, or DMS (6). They apply their three criteria and conclude that any claims that signs of life have been detected fails each test. One must be careful to remember that in ref. 6 only the detection of DMS has been claimed, not that it necessarily points to biology (although as a possibility this is mentioned in the paper). Seager et al. point to two solar system cases as analogous—phosphine on Venus (13) and methane at the Martian surface (14), although the latter detection is robust.

These controversies arise because of the irresistible desire of scientists to use their instruments at the very limits of their capabilities. Such a desire is understandable: Science is, after all, about expanding knowledge and doing so requires ever better instruments, the imperative and design of which come from pushing existing instruments to their limits. In the case of JWST, beautiful transit spectra with good S/N of planets down to the size even of Neptune are reliably obtained. Spectra this good for Earth-sized planets around Sun-like stars must await the Habitable Worlds Observatory, at the earliest 20 years in the future. Scientists can and should use powerful tools like JWST to the maximum extent possible, including various difficult targets such as K2-18b. But in doing so, one must be mindful of the words of Carl Sagan (15).

*“By and large, scientists’ minds are open when exploring new worlds. If we knew beforehand what we’d find, it would be unnecessary to go…surprises—even some of mythic proportions—are possible, maybe even likely. But we humans have a talent for deceiving ourselves. Skepticism must be a component of the explorer’s toolkit, or we will lose our way. There are wonders enough out there without our inventing any.”*

## Data Availability

All data referred to in this paper are from cited articles in the published literature.

## Footnotes

^*^“Sono dumque soli innumerabili, sono terre infinite che similmente circuiscono que’ soli; come veggiamo questi sette circuire questo sole a noi vicino... noi veggiamo gli soli, che son gli più grandi, anzi grandissimi corpi: ma non veggiamo le terre, le quali per esserno corpi molto minori, sono invisibili.” This is a more literal translation than what one often sees on the web.
